# Are measurements of systolic myocardial velocities and displacement with colour and spectral Tissue Doppler compatible?

**DOI:** 10.1186/1476-7120-7-29

**Published:** 2009-06-23

**Authors:** Aristomenis Manouras, Arben Shala, Evangelia Nyktari, Kambiz Shahgaldi, Reidar Winter, Panagiotis Vardas, Lars-Åke Brodin, Jacek Nowak

**Affiliations:** 1 Department of Clinical Physiology, Karolinska University Hospital in Huddinge, Stockholm, Sweden; 2 Department of Cardiology, Karolinska University Hospital in Huddinge, Stockholm, Sweden; 3 School for Technology and Health, Royal Institute of Technology, Flemingsberg, Stockholm, Sweden; 4 Department of Cardiology, University Hospital of Crete, Heraklion, Greece

## Abstract

**Background:**

Tissue Doppler (TD) in pulsed mode (spectral TD) and colour TD are the two modalities today available in tissue velocity echocardiography (TVE). Previous studies have shown poor agreement between these two methods when measuring myocardial velocities and displacement. In this study, the concordance between the myocardial velocity and displacement measurements using colour TD and different spectral TD procedures was evaluated.

**Methods:**

Left ventricular (LV) longitudinal systolic myocardial velocities and displacement during ejection period were quantified at the basal septal and lateral wall in 24 healthy individuals (4 women and 20 men, 34 ± 12 years) using spectral TD, colour TD and M-mode recordings. Mean, maximal and minimal spectral TD systolic velocities and the corresponding displacement values were obtained by measurements at the outer and inner borders of the spectral velocity signal. The results were then compared with those obtained with the two other modalities used.

**Results:**

Systolic myocardial velocities derived from mean spectral TD frequencies were highly concordant with corresponding colour TD measurements (mean difference 0.10 ± 0.54 cm/sec in septal and 0.09 ± 0.97 cm/sec in lateral wall). Similarly, the agreement between spectral and colour TD (mean difference 0.22 ± 0.74 mm in septal and 0.02 ± 0.86 mm in lateral wall) as well as M-mode was good when mean spectral velocities were temporally integrated and the results did not differ statistically. Conversely, displacement values from the inner or outer border of the spectral signal differed significantly from values obtained with colour TD and M-mode (p < 0.001, in both cases).

**Conclusion:**

LV systolic myocardial measurements based on mean spectral TD frequencies are highly concordant with those provided by colour TD and M-mode. Hence, in order to maintain compatibility of the results, the use of this particular spectral TD procedure should be advocated in clinical praxis.

## Background

Introduction of tissue velocity echocardiography (TVE) two decades ago[1] has opened new possibilities for non-invasive quantification of myocardial function. TVE methodology relies on the detection of the low velocity/high amplitude motion of myocardial tissue by application of appropriate low pass filtering to the received Doppler signal in order to distinguish it from the high velocity/low amplitude motion of the blood [2,3]. The diagnostic performance of TVE has been extensively studied and its incremental value for the evaluation of hypertrophic cardiomyopathy [4], coronary artery disease [5,6], and the determination of systolic and diastolic left ventricular function [7,8] is well documented.

Tissue Doppler in pulsed mode (pulsed/spectral TD) [9,10] and colour TD [11,12] are the two modalities available today in tissue velocity echocardiography (TVE). Spectral TD registers the instantaneous frequency spectrum at the chosen myocardial region and the signal is computed using Fast Fourier Transform (FFT) technique in order to obtain the myocardial velocity distribution. On the other hand, colour TD relies on autocorrelation analysis to provide average Doppler frequencies for a chosen set of pixels and thus yields mean myocardial velocities in the interrogated myocardial region. Both TVE modalities have been validated in experimental [13,14] and clinical studies [9,11,15] and the requirements for optimal signal sampling [16] as well as the effect of instrumentation settings on the accuracy of TD measurements [17,18] have been evaluated.

However, despite the fact that the two tissue Doppler modalities target the same physical quantity i.e. velocity, they operate in different ways and, indeed, a number of comparative studies performed hitherto have shown poor agreement between the results obtained with the two modalities [17,19,20]. This is not surprising, since the general applied method of myocardial velocity estimation with spectral TD involves measurements performed at the outer border of the spectral velocity wave. Consequently, this procedure identifies maximal components of the velocity spectrum in the region of interest, whereas with colour TD modality, a mean velocity at the interrogated myocardial location is obtained. Hence, it appears reasonable to assume that spectral TD velocity measurements based on averaged, rather than maximal signal, would better agree with the results of corresponding measurements obtained with colour TD technique. Since, to our knowledge, this issue has not yet been specifically addressed as yet, the aim of this study was to compare the results of myocardial velocity and displacement measurements using colour TD with those obtained with spectral TD based on averaging of the spectral signal.

## Methods

The study involved 24 healthy volunteers without any known cardiovascular disease (4 women and 20 men; 34 ± 12 years). The study protocol was approved by the ethics committee of Karolinska University Hospital, Stockholm Sweden.

### Tissue velocity echocardiography

Tissue velocity echocardiography was performed using commercially available equipment (Vivid 7, GE Vingmed, Horten, Norway) with a standard phased array 2.5 MHz multi-frequency transducer. The images were acquired from apical four chamber and two chamber views with the patient in left lateral position, at the end of expiration. All patients were in sinus rhythm. Cineloops of at least 3 heartbeats were acquired with high temporal resolution (frame rate range: 270 ± 28 Hz) and stored digitally for subsequent off-line analysis. The stored raw data containing grey-scale and colour tissue TD as well as spectral TD velocity information was analysed using Echopac software (version 6.0.0, GE Vingmed Ultrasound, Norway).

#### Colour TD

**Figure 1:**
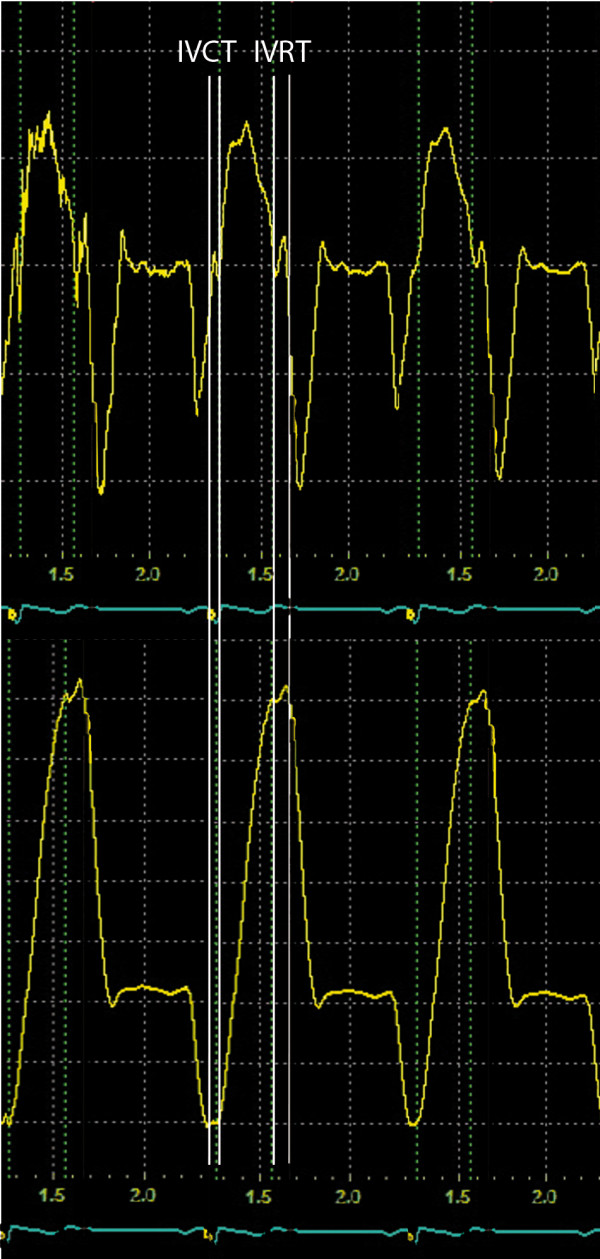
**Typical LV longitudinal myocardial velocity curves *(top) *obtained with colour TD from the basal segment of septum (*insertion*)**. Integration of the velocity curves over time yielded the corresponding longitudinal myocardial displacement curves *(bottom)*. Isovolumic contraction time (IVCT) and isovolumic relaxation time (IVRT) are indicated.

The employed Echopac software allows real-time digital acquisition of tissue velocity curves at any chosen point within the myocardium. The obtained velocity curves (Figure 1, top) can then be integrated over time yielding the corresponding myocardial displacement curves (Figure 1, bottom). The analysis of LV myocardial velocities and displacement was performed from the measuring point set at the septal and lateral LV segment just below the level of mitral annulus using a 6 × 6 pixels sampling volume. Maximal systolic velocity (colour PSV) was initially measured. Subsequently, without changing the positioning of the region of interest, LV displacement during the systolic ejection (SE_m_d) was obtained by measuring the amplitude of the LV myocardial displacement curve (Figure 1, bottom) during systolic ejection. Isovolumic contraction and relaxation phases were excluded. All measurements were performed on 3 cardiac cycles and averaged. No temporal filtering was applied.

The timing of the cardiac electromechanical events was determined using the method proposed by Lind et al. [21]. The opening of the aortic valve was thus defined as occurring at the zero crossing point of the ascending limb of the myocardial velocity wave at the beginning of systolic ejection, whereas the zero crossing point for the descending limb of systolic ejection wave at the end of systolic ejection marked the aortic valve closure. A clear zero-crossing point was identified in all recordings.

#### Spectral TD

While keeping the image sector width unaltered and thus the frame rate the same as for colour TD recordings, spectral TD data were acquired from the measuring point set immediately below the mitral ring at the respective basal septal and lateral LV segment using a 5.9 mm sample volume. Special care was taken to keep the sampling volume positioned continuously on the ventricular myocardium, with insonation angle as parallel as possible with the long axis of systolic myocardial movement. Depth and Nyquist limit were adjusted and kept unaltered during each examination. The pulse repetition frequency was higher than 960 Hz (1072 ± 211) in all spectral registrations. The manufacturer's default transmit gain of 0 dB and receive gain of 3 dB were used.

**Figure 2:**
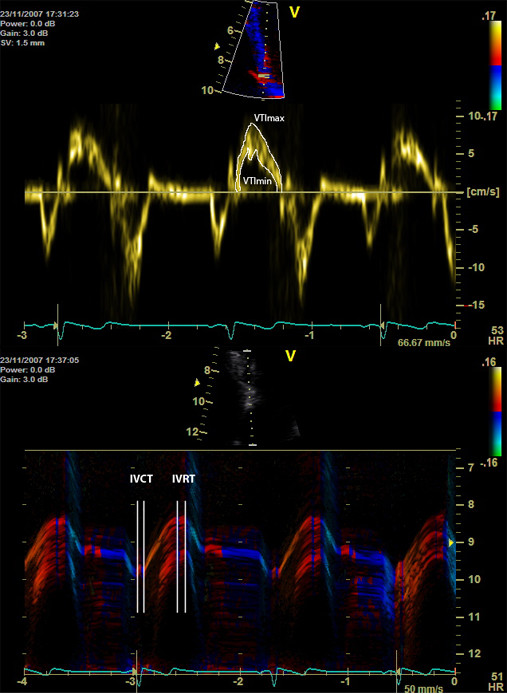
***(Top) *Typical LV longitudinal myocardial velocity curves obtained with spectral TD from the most basal segment of septum (*insertion*)**. The respective curves were traced at the outer and the inner border of the velocity waveform and mean velocity was calculated by averaging spectral maximal and minimal velocity. The respective curves were integrated over time to yield VTI_max _and VTI_min_. Mean velocity time integral (VTI_mean_) was determined by averaging VTI_max _and VTI_min_. *(Bottom) *Typical colour-coded M-mode image of LV longitudinal myocardial motion acquired at basal septum used for the measurements of systolic displacement. Boundaries of isovolumic contraction (IVCT) and relaxation (IVRT) periods were established by observing the specific colour bands expressing the reversal of velocity direction at the beginning and the end of systole [21].

The spectral TD velocity signals were traced offline at the outer and inner border of the pulsed TD velocity wave as proposed by Chen et al. [22] and the highest (spectral SV_max_) and lowest (spectral SV_min_) systolic myocardial velocities were thus determined. The respective velocity time integrals representing maximal (VTI_max_) and minimal (VTI_min_) systolic myocardial displacements were subsequently calculated (Figure 2, top). Next, the mean velocity (spectral SV_mean_) was calculated by averaging spectral SV_max _and SV_min _and mean time integral (VTI_mean_) was determined by averaging VTI_max _and VTI_min_. The isovolumic events were excluded from the measurements. Their respective starting and ending points were defined using the same procedure as described above for colour TD [21]. The tracings with uneven spectral envelope contour were excluded from the measurements. Measurements obtained from 3 cardiac cycles were averaged.

### M-mode echocardiography

M-mode recordings were performed at septal and lateral margins of mitral annulus on colour TD superimposed 2 D images keeping the same image sector width as for colour and spectral TD recordings (Figure 2, bottom). Temporal resolution employed was higher than 1000 Hz (1211 ± 226) for all performed measurements. Care was taken in order to position the cursor parallel to the longitudinal axis of wall motion. Colour TD superimposition on M-mode recordings provides the possibility for accurate off-line measurements with delineation of the electromechanical cardiac events. The onset of the systolic ejection phase coincides with the beginning of the steep upward myocardial motion in M-mode tracing and the start of a broad red band at colour TD -coded M-mode. The closure of aortic valve is identified at the red-blue band interface as suggested by Lind et al. [21] (Figure 2, bottom). Mitral annulus motion during systolic ejection phase (MAM) was measured excluding atrioventricular motion during isovolumic phases. Measurements were performed at 3 cardiac cycles and averaged.

### Statistical analysis

All data are presented as mean ± SD unless otherwise stated. The statistical significance level was set at p < 0.05. Group comparisons of continuous variables were made using analysis of variance (ANOVA) followed by post hoc Scheffé's test. Pearson's correlation coefficient was used for analysis of linear correlation between the results of the evaluated methods. Student's t-test was used when suitable for comparisons of paired data. The analyses were carried out using standard statistical software (Statistica, version 8.0). Methodological error (Err) in a single measurement estimated from double measurements was calculated according to formula: Err = (SD_diff _× 100%)/(total mean x√2), where SD_diff _is the SD of the difference between the measurements. Assessment of the agreement in velocity and displacement measurements was performed using the method of Bland and Altman [23].

## Results

### Myocardial velocities

The LV myocardial longitudinal systolic velocity values obtained with colour- and spectral TD are presented in Additional file 1. As can be seen from the table, at both interrogated wall segments, the systolic myocardial tissue velocities traced at the outer and inner border of the velocity spectrum differed significantly not only from the colour TD generated peak systolic velocities but also from the mean spectral velocity obtained by averaging spectral SV_max _and SV_min_. On the other hand, no significant difference occurred between PSV measured with colour TD and mean spectral velocity obtained with spectral TD.

**Figure 3:**
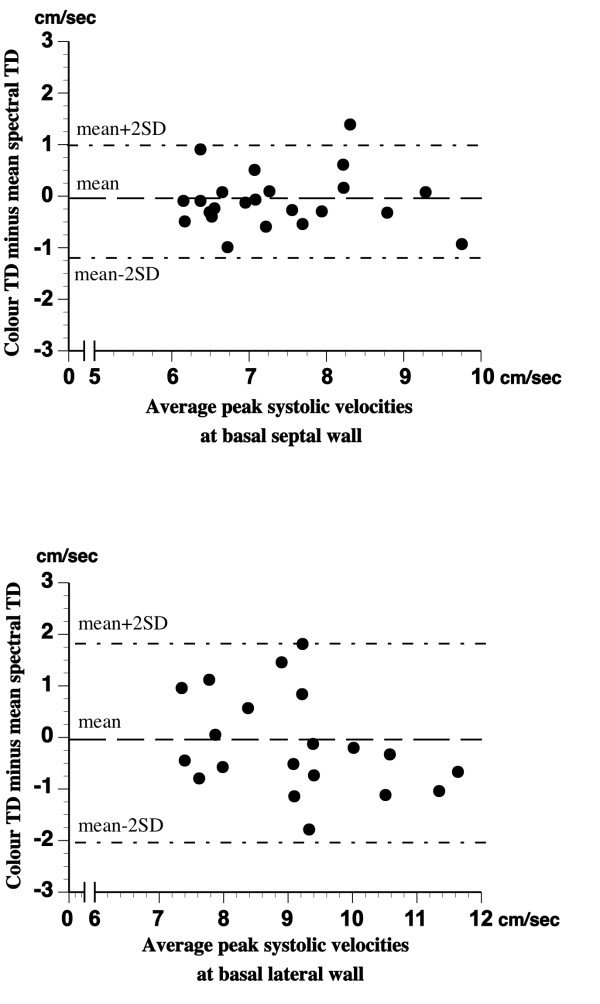
**Bland-Altman plot of differences between LV myocardial longitudinal systolic velocities measured by colour TD and mean spectral TD procedure at the basal septal *(top) *and basal lateral *(bottom) *wall**.

The results of LV myocardial systolic velocity measurements by spectral TD correlated well (p < 0.001, in all cases) with those produced by colour TD technique (Additional file 2). However, despite the observed significant relationships, the poor agreement between the measurements performed at the outer and inner border of the tissue velocity spectrum and those obtained by colour TD is evident from the results of Bland Altman analysis presented in Additional file 2. As can be seen, the spectral TD SV_max _overestimated, whereas spectral TD SV_min _underestimated colour TD PSV by a mean value of 1.52 cm/sec and 1.32 cm/sec, respectively, and the limits of agreement for the measured variables were particularly wide in lateral wall. On the other hand, when spectral TD SV_mean _was calculated by averaging SV_max _and SV_mean_, the obtained results were almost identical to those provided by colour TD and the limits of agreement were clearly narrower (figure 3).

The errors of the double measurements of LV systolic velocities performed at septal wall by two independent observers employing colour TD as well as the three different spectral TD procedures are presented in Additional file 3. As can be seen, the error of spectral measurements was twice as big as that of colour TD determinations.

### Myocardial displacement

The mean values of LV myocardial displacement obtained by using M-mode, colour TD, and the three different spectral TD procedures are presented in Additional file 4. Similar to what was observed for myocardial velocities, the velocity time integrals obtained with the maximal and minimal spectral velocities differed significantly (p < 0.001, in all cases) from the displacement values produced by M-mode, colour TD, and from systolic time integral of mean spectral velocity. No statistically significant differences occurred between M-mode, colour TD and spectral VTI_mean _in septal wall whereas in lateral wall, M-mode generated displacement values were somewhat higher than those obtained with colour TD and the spectral VTI_mean _method (p < 0.05, in both cases).

**Figure 4:**
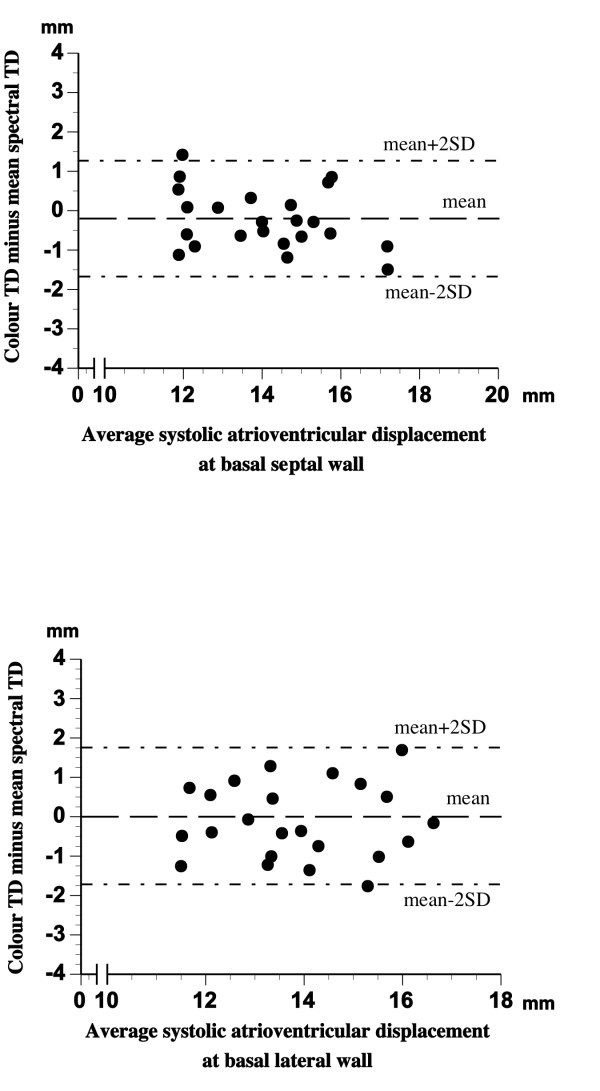
**Bland-Altman plot of differences between LV myocardial longitudinal systolic displacement measured by colour TD and mean spectral TD procedure at basal septal *(top) *and basal lateral *(bottom) *wall**.

**Figure 5:**
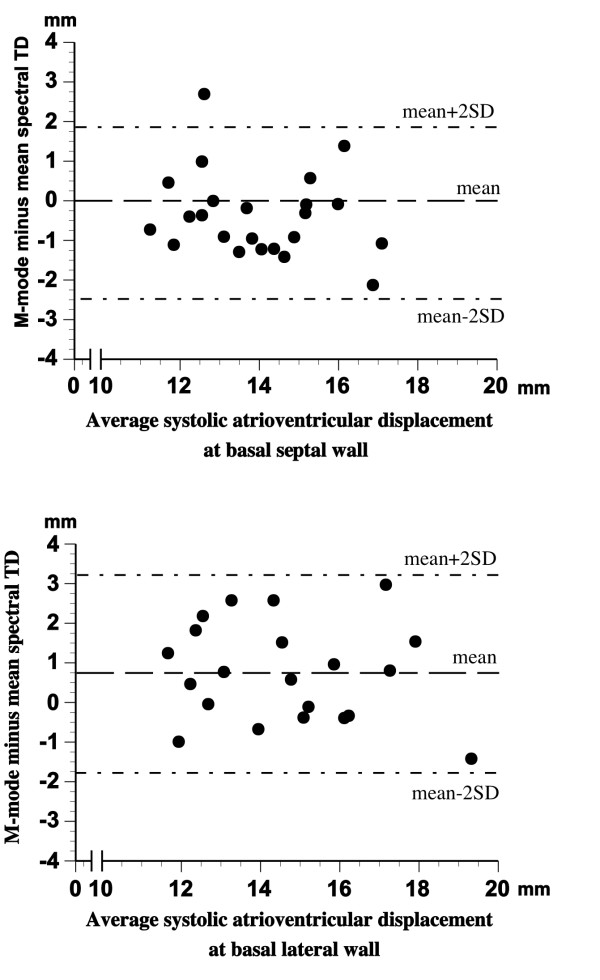
**Bland-Altman plot of differences between LV myocardial longitudinal systolic displacement measured by M-mode and mean spectral TD procedure at basal septal *(top) *and basal *(lateral) *wall**.

**Figure 6:**
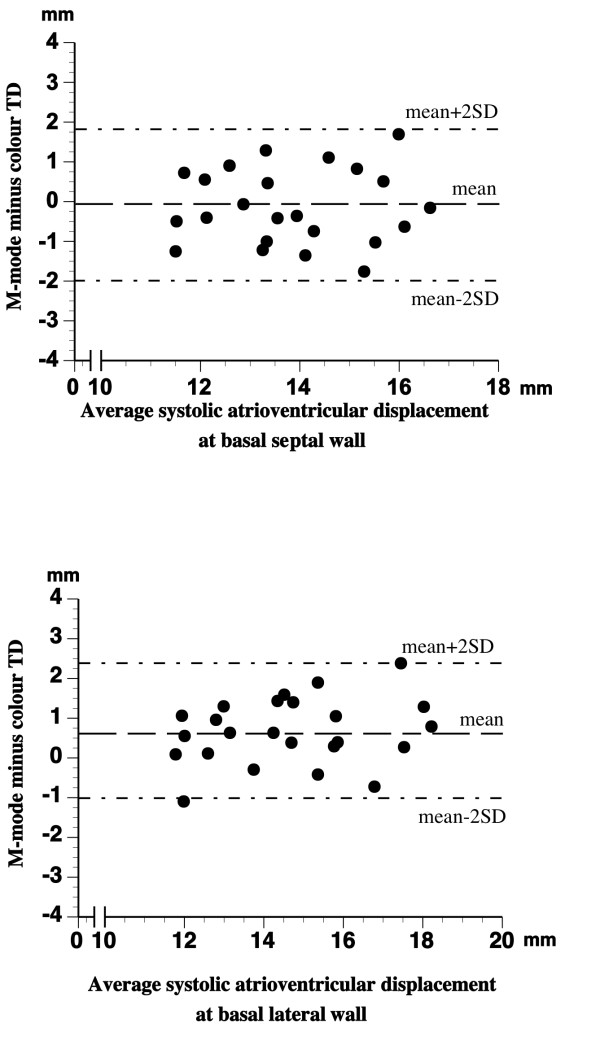
**Bland-Altman plot of differences between LV myocardial longitudinal systolic displacement measured by M-mode and colour TD at basal septal *(top) *and basal lateral *(bottom) *wall**.

There was a significant relationship (p < 0.001, in all cases) between the results of LV myocardial systolic displacement measurement with M-mode, colour TD, and the three spectral TD procedures (Additional file 5). However, as can be seen from the results of Bland Altman analysis presented in Additional file 5, the agreement between VTI_max _and VTI_min _on one side and the colour TD and M-mode produced displacement values on the other was rather poor, with the least mean difference between the methods of over 3.6 mm (M-mode vs. spectral VTI_min_) and wide limits of agreement in all cases. On the other hand, the results of spectral VTI_mean _measurements agreed well with the displacement values obtained with colour TD and M-mode within narrow limits of agreement (figure 4 and 5). The best combination of mean differences and limits of agreement in septal and lateral LV wall was found for spectral VTI_mean _and colour TD displacement measurements (see Additional file 5 and figure 4). There was a good agreement between the results of M-mode and colour TD displacement measurements as well (figure 6).

The errors of the double measurements of LV systolic myocardial displacement performed at septal wall by two independent observers employing M-mode, colour TD, and the three different spectral TD procedures are presented in Additional file 3.

## Discussion

The present results demonstrate that longitudinal systolic myocardial velocities derived from colour TD are not only closely correlated but also in good agreement with mean spectral TD velocities. A similar degree of concordance and correlation was observed for LV systolic myocardial displacement measured by colour TD and mean spectral TD procedure. This is not entirely unexpected when considering the physical principles of the two TVE techniques. Indeed, regarding spectral TD, the acquired composite frequency signal is subjected to Fast Fourier analysis and the sum of velocity distributions from the individual scatterers passing through the range gate is obtained at time intervals defined by the pulse repetition frequency. The spectral signal reflects thus the sum of velocity distributions at any instant during the recording period. Assuming Gaussian distribution of instantaneous myocardial velocities at the interrogated region [24], mean velocities are to be found in the mid portion of the spectral signal while the outer and inner contours of the envelope reflect maximal and minimal myocardial velocities, respectively. Colour TD, on the other hand, relies on autocorrelation algorithm in order to estimate the average frequency shift for a given set of pixels along the Doppler sector and provides consequently mean myocardial velocities at the region of interest. Hence, it can be expected that colour TD and mean spectral TD velocities would closely relate to each other.

To our knowledge, there are no studies published hitherto that compare directly colour and mean spectral TD velocities. Recently, Chen et al. have reported that systolic as well as early diastolic longitudinal velocities measured at the mid portion of the spectral signal were nearly equivalent to those obtained by digitized M-mode recordings at septal and lateral site of mitral annulus [22]. Our findings add to these observations and demonstrate that colour TD and mean spectral TD procedure provide almost identical values when measuring systolic velocities. However, the results of myocardial velocity measurements with colour TD and mean spectral TD procedure may vary between -2.03 and 1.85 cm/sec (lateral wall), a fact that should be kept in mind in the clinical setting, especially when low longitudinal velocities are recorded as in the case of ischemic myocardium. The slight non-significant numerical underestimation of mean spectral velocities by colour TD can, at least partly, be explained by the higher inherent temporal resolution of pulsed TD. Despite the unusually high frame rate employed in this study for colour TD recordings (>250 Hz) and the fact that measurements were performed on unfiltered data, the temporal resolution obtained was still well below that of spectral TD recordings and this might result in a possible underestimation of minor myocardial motions. Furthermore, Walker et al. demonstrated that spectral TD tended to overestimate tissue velocities [25], an observation well in accord with the present findings.

The current results reveal that colour TD and mean spectral TD myocardial velocities are concordant even when temporally integrated. While the employed software provides a computerized temporal integration of colour TD velocities in order to assess myocardial displacement, in the case of spectral TD, manual tracing of velocity spectra is required for estimation of myocardial motion. Nevertheless, no statistical differences between the displacement measurements performed with colour TD and mean spectral TD procedure appeared between the two interrogated myocardial walls. Still, our results imply that displacement values measured by the two TVE modalities may differ as much as 4 mm (lateral wall), which should be considered in the clinical situation.

Furthermore, similar to what was observed in the study of Lind et al. [26], the present M-mode measurements of mitral annulus motion at the lateral wall produced displacement values that were significantly higher than those obtained with colour TD and mean spectral TD method. The overestimation of colour TD displacement by M-mode on the lateral wall was recently confirmed by Ballo et al. [27] and the authors reported that the agreement between M-mode and spectral TD measurements was improved when adjusted (mean) spectral TD signal was used. The present results are in keeping with these observations even if the discrepancy between M-mode and colour TD measurements in the present study was less pronounced, possibly as a result of the higher colour TD sampling frequency currently used.

The overestimation of both colour and mean spectral TD displacement at the lateral wall site observed by M-mode may be partly the result of an unfavourable angle of incidence. Indeed, an optimal insonation angle is difficult to be achieved at the lateral wall site. For TVE measurements, deviation of the incidence angle would lead to underestimation of longitudinal myocardial velocities and displacement and the underestimation would be directly dependent on the cosine of divergence angle of the Doppler beam from the longitudinal plane. On the other hand, an unfavourable insonation angle of the M-mode beam would result in overestimation of the true longitudinal motion due to the influence of concomitant radial myocardial movement as the mean vector of radial and longitudinal motion is measured.

The lower interobserver variability for current spectral measurements compared with what was previously reported [15,28] reflects most probably standardization of the present TD recordings and exclusion of signals with uneven spectral envelope contour. However, it still reflects the subjectivity in identifying the borders of spectral envelope. The reproducibility of the present colour TD velocity measurements was better than that of spectral TD and similar to that previously documented in a larger study [29]. Furthermore, in order to approximate the high inherent repetition frequency of the spectral TD and accurately register minor myocardial motions, colour TD recordings were performed employing high frame rates without applying temporal filtering on off-line measurements [16,18]. However, increased temporal resolution may as well yield an increased noise to signal ratio which in turn may influence the results obtained with colour TD analysis. The interobserver variability values obtained in the present study was similar to those reported previously [29] and any significant confounding influence of high sampling frequency appears thus to be unlikely. Finally, the number of individuals included in this study was rather small and limited on healthy volunteers. Larger studies on consecutive groups of patients are needed in order to conclusively confirm the present results and the interchangeability of colour TD and spectral TD procedure.

Up to date, specific technical and measuring recommendations for spectral measurements are lacking. It is generally recommended that gain settings should be adjusted at a minimal level in order to avoid spectral broadening but yet the technical requirements are not specified which may, at least partly, explain the discrepancy observed between different TD studies. Indeed, while significant correlation between systolic myocardial velocities measured with pulsed TD and left ventricular ejection fraction has been found in a number of studies [30,31], other reports based on colour TD show only weak association between these two indices [29]. In the present study, spectral recordings were performed applying transducer settings conventionally used as default by the manufacturer with transmit gain of 0.0 dB, receive gain of 3.0 dB and off-line gain saturation 50% saturation.

Regardless the aforementioned limitations, the results of the present study have some important clinical implications. Chen et al. in their study have shown that mid envelope (mean) spectral velocities were almost identical with velocities measured with digitized M-mode. The authors concluded that their findings together with the lack of standardized measuring conventions for spectral TD recordings could imply that mean spectral TD procedure may be recommended in clinical routine [22]. However, the application of mean spectral TD method, especially for displacement measurements, is rather laborious and time consuming which may jeopardize the use of this method in clinical praxis. On the other hand, based on the present results showing that colour TD provides velocity and displacement measurements that are in good agreement with mean spectral TD and M-mode calculations, the use of colour TD with sufficiently high temporal resolution may be recommended as an efficient and reliable tool for quantification of myocardial function in clinical situation.

## Conclusion

Quantification of systolic and diastolic myocardial function using tissue Doppler velocity profiles is today an established and broadly used echocardiographic technique. However, the rapidly growing use of both pulsed and colour TD modalities revitalises the issue of occasional ambiguity of the results obtained and of parallel use of these methods in clinical practise. Against this background, the current results are of significance since they demonstrate that LV systolic myocardial measurements based on mean spectral TD frequencies are highly concordant with those provided by colour TD and M-mode. Hence, if spectral TD is to be employed, the use of the mean spectral TD procedure should be advocated. The present results underscore at the same time the importance of standardization of spectral TD procedure, if the results obtained by M-mode, colour TD and spectral TD methods are to be considered compatible. However, the recently reported high sensitivity of spectral TD to gain settings [17,32] may favour the preferential use of colour TD in clinical praxis.

## Competing interests

The authors declare that they have no competing interests.

## Authors' contributions

AR contributed to the design of the study, performed measurements and calculations from ultrasound data as well as statistical analysis, and participated in the interpretation of the results and preparation of the manuscript. AS performed measurements and calculations from ultrasound data as well as statistical analysis. EN participated in the interpretation of the results and preparation of the manuscript. PV contributed to the interpretation of the obtained results. KS participated in data collection and interpretation of the results. RW and LÅB supervised the study and contributed to the interpretation of the obtained results. JN supervised the study, contributed to the analysis and interpretation of the data, and was responsible for the preparation of the final version of the manuscript. All authors read and approved the final manuscript.

## Supplementary Material

Additional file 1 **Table 1. ** Systolic myocardial velocities (cm/sec) obtained by colour and spectral TD at septal and lateral wall. The data presented provide myocardial longitudinal systolic velocities measured with colour and spectral TD at the septal and lateral wall site of mitral annulus. Click here for file

Additional file 2 **Table 2. **Mean differences and limits of agreement (mean ± 2SD) for colour TD and spectral TD systolic velocities (cm/sec) at septal and lateral wall. The data presented give mean values and limits of agreement for myocardial systolic velocities measured with colour TD and spectral TD at the outer and the inner border of the spectral envelope as well as with the mean spectral TD procedure. Click here for file

Additional file 3 **Table 3. ** Interobserver variability of colour and spectral TD derived myocardial velocity and displacement values at septal wall. The data presented give the interboserver variability values (%) for myocardial systolic velocity and displacement measurements obtained with colour TD, M-mode, as well as the three different spectral TD Doppler procedures at the basal septal wall site of mitral annulus. Click here for file

Additional file 4 **Table 4. **LV myocardial longitudinal displacement (mm) during systolic ejection obtained by M-mode, colour and spectral TD at septal and lateral wall. The data presented provide myocardial longitudinal systolic displacement values at the basal septal and lateral wall site measured by colour TD, M-mode and different spectral TD procedures. Click here for file

Additional file 5 **Table 5. ** Mean differences and limits of agreement (mean ± 2SD) for colour TD, spectral TD and M-mode measurements of LV myocardial longitudinal displacement during systolic ejection.The data presented give mean differences and limits of agreement for myocardial longitudinal systolic displacement values at the basal septal and lateral wall site measured by colour TD, M-mode and different spectral TD procedures. Click here for file
